# CXCL2-CXCR2 axis mediates αV integrin-dependent peritoneal metastasis of colon cancer cells

**DOI:** 10.1007/s10585-021-10103-0

**Published:** 2021-06-11

**Authors:** Mattias Lepsenyi, Nader Algethami, Amr A. Al-Haidari, Anwar Algaber, Ingvar Syk, Milladur Rahman, Henrik Thorlacius

**Affiliations:** Section of Surgery, Department of Clinical Sciences, Malmö, Skåne University Hospital, Lund University, 20502 Malmö, Sweden

**Keywords:** Chemokines, Chemotaxis, Integrins, Peritoneal carcinomatosis, Metastasis

## Abstract

Peritoneal metastasis is an insidious aspect of colorectal cancer. The aim of the present study was to define mechanisms regulating colon cancer cell adhesion and spread to peritoneal wounds after abdominal surgery. Mice was laparotomized and injected intraperitoneally with CT-26 colon carcinoma cells and metastatic noduli in the peritoneal cavity was quantified after treatment with a CXCR2 antagonist or integrin-αV-antibody. CT-26 cells expressed cell surface chemokine receptors CXCR2, CXCR3, CXCR4 and CXCR5. Stimulation with the CXCR2 ligand, CXCL2, dose-dependently increased proliferation and migration of CT-26 cells in vitro. The CXCR2 antagonist, SB225002, dose-dependently decreased CXCL2-induced proliferation and migration of colon cancer cells in vitro. Intraperitoneal administration of CT-26 colon cancer cells resulted in wide-spread growth of metastatic nodules at the peritoneal surface of laparotomized animals. Laparotomy increased gene expression of CXCL2 at the incisional line. Pretreatment with CXCR2 antagonist reduced metastatic nodules by 70%. Moreover, stimulation with CXCL2 increased CT-26 cell adhesion to extracellular matrix (ECM) proteins in a CXCR2-dependent manner. CT-26 cells expressed the αV, β1 and β3 integrin subunits and immunoneutralization of αV abolished CXCL2-triggered adhesion of CT-26 to vitronectin, fibronectin and fibrinogen. Finally, inhibition of the αV integrin significantly attenuated the number of carcinomatosis nodules by 69% in laparotomized mice. These results were validated by use of the human colon cancer cell line HT-29 in vitro. Our data show that colon cancer cell adhesion and growth on peritoneal wound sites is mediated by a CXCL2-CXCR2 signaling axis and αV integrin-dependent adhesion to ECM proteins.

**Supplementary Information:**

The online version contains supplementary material available at 10.1007/s10585-021-10103-0.

## Introduction

Colorectal cancer is the third leading cause of cancer-related death worldwide and mortality is generally related to cancer cell metastasis [1]. Peritoneal carcinomatosis is considered to be present in around 10% of patients with colorectal cancer at some point during the course of the disease [2–4]. The occurrence of peritoneal metastasis is believed to be a result of penetrating growth of the primary tumor allowing shedding of malignant cells intraperitoneally or a consequence of handling during surgery when tumor site, lymphatics or blood vessels are physically traumatized. Colorectal peritoneal carcinomatosis has for a long time been considered to be a terminal condition until the late nineties, when cytoreductive surgery (CRS) with peritonectomy together with hyperthermic intraperitoneal chemotherapy (HIPEC) was described and popularized as a potential option for patients with peritoneal carcinomatosis [5]. CRS combined with HIPEC is the only treatment that has shown curative potential on peritoneal carcinomatosis of colorectal origin with reported five-year survival rates of 35–40% in large series [6–8]. However, peritoneal carcinomatosis recurrence rate is still high (40–60%), which underlines the need for developing new routes of action to prevent tumor establishment and growth in the peritoneal cavity [9]. Thus, increased understanding of the mechanisms promoting peritoneal dissemination of colon cancer cells could help improving the efficacy of CRS/HIPEC treatment of patients with peritoneal carcinomatosis.

Convincing data suggest that chemokines regulate multiple aspects of tumor cell biology, including survival, proliferation, angiogenesis and migration [10, 11]. Chemokines belong to a superfamily of small molecules (8–14 kDa) that were initially discovered due to their interactions with chemokine receptors which regulate leukocyte trafficking to sites of inflammation [12, 13]. Numerous studies have shown that chemokine receptors, such as CXCR2, are expressed on colon cancer cells [14–16] and has been shown to support hepatic metastasis of colon cancer [17–19]. In this context, it is interesting to note that the CXCR2 ligand CXCL2, is an important pro-inflammatory mediator and a powerful chemoattractant for neutrophils, which is up-regulated during wound healing [20, 21]. Besides accumulation at sites of wound healing, adherence to exposed extracellular matrix (ECM) is essential for cell survival and growth [22]. Different types of integrins constitute receptors for ECM proteins. Integrins are heterodimeric proteins of noncovalently bound subunits that are expressed on the cell surface [23]. αV integrins are known to facilitate tumor cell adhesion to ECM [24]. There are five types of αV integrins, i.e. αVβ1, αVβ3, αVβ5 and αVβ6 and αVβ8 and the main ligands of αV integrins are vitronectin, fibronectin and fibrinogen [25]. The role of CXCR2 and αV integrins in the peritoneal dissemination of colon cancer cells remain elusive.

Based on the above, the aim of this study was to define the mechanisms of colon cancer spread and accumulation at peritoneal wounds in vivo. For this purpose, we used a murine model with laparotomy causing a midline incisional wound simulating abdominal surgery for colorectal cancer and intraperitoneal administration of colon cancer cells mimicking free disseminated cancer cells.

## Materials and methods

### Cells and reagents

The murine colon adenocarcinoma cell line, CT-26 transfected with green fluorescent protein (GFP), was a kind gift from Dr Otto Kollmar (University of Saarland, Germany) and human epithelial colon adenocarcinoma cell line, HT-29, was obtained from American Type Culture Collection (HTB-38, ATCC, Manassas, VA, USA). Cells were cultured in Roswell Park Memorial Institute medium (RPMI, Sigma-Aldrich, Stockholm, Sweden), supplemented with 10% FBS, 2 mM L-glutamine, 10 000 units penicillin and 10 mg streptomycin/mL at 37 °C and 5% CO2. Calcein AM, CXCR2 antagonist; N-(2-Hydroxy-4-nitrophenyl)-N′-(2-bromophenyl) urea (SB225002), and accutase were obtained from Sigma-Aldrich. Recombinant mouse CXCL2 was purchased from Peprotech (Rocky Hill, NJ, USA). PE-labeled anti-mouse CXCR2, APC-labelled anti-mouse CXCR3, β1 integrin, β3 integrin and CXCR4, PE-Cy7-labelled anti-mouse CXCR5, blocking anti-human CD51/61 (αVβ3) antibody, FITC-labeled anti-human CXCR1, PE-labelled anti-human CXCR2, FITC-labeled anti-human CXCR3 and APC-labeled anti-human CD51/61 (αVβ3) were purchased from BioLegend (San Diego, CA, USA). PE-labelled anti-mouse αV integrin and recombinant human IL-8/CXCL8 protein were purchased from R&D systems Europe (Abingdon, UK). Blocking anti-mouse αV antibody was purchased from Affymetrix (Santa Clara, CA, USA). Anti-mouse Fc block CD16/32 antibody was obtained from eBioscience (San Diego, CA, USA). The CXCR1/CXCR2 antagonist (Reparixin) was purchased from TOCRIS (Bristol, UK).

### Flow cytometry analysis of cell surface receptors

Surface expression of chemokine receptors and integrins were assessed using flow cytometry. CT-26 and HT-29 cells were detached with accutase when reaching 80% confluence. To reduce nonspecific antibody binding via Fc receptors, samples were incubated with 1 μg of anti-mouse CD16/CD32 antibody or human Fc receptor blocking antibody for 15 min, respectively. Thereafter, CT-26 cells were incubated with 1 μg of PE-labelled anti-mouse CXCR2, APC-labelled anti-mouse CXCR3, CXCR4, β1 integrin and β3 integrin, PE-Cy7-labelled anti-mouse CXCR5 or PE-labelled anti-mouse αV integrin in separate tubes. While HT-29 cells were incubated with 1 μg of FITC-labeled anti-human CXCR1 and CXCR3, PE-labeled anti-human CXCR2 or APC-labeled anti-human αVβ3 were incubated at room temperature for 20 min. Cells were then washed twice and resuspended in 0.4 ml final volume of FACS buffer and analysed using cytoflex (Beckman Coulter, Mountain View, CA, USA). Unstained cells served as negative control. Histograms were made using CytExpert software with assessment of 10,000 events per sample.

### Chemotaxis assay

Chemotactic response of CT-26 and HT-29 cells was evaluated by using 24-well cell migration chambers with 8 μm pore size inserts (Corning Coster, Corning, NY, USA). Both CT-26 and HT-29 cells were serum starved overnight and resuspended in serum-free DMEM with 0.5% BSA and 5 × 10^5^ cells/ml were loaded in the inserts. CT-26 migration assay was tested by adding DMEM with or without 50, 100, and 200 ng/ml of CXCL2 in the lower chambers. In separate experiments, HT-29 cell migration assay was studied by adding DMEM with or without 10, 50, 100 ng/ml of CXCL8 in the lower chamber. Assays were incubated for 24 h (37 °C, 5% CO_2_). Non-migrated cells were removed by cotton swabs from the upper surface of the insert and cells on the lower surface of the insert membrane were fixed in ice-cold 100% methanol and stained with 1% crystal violet. In certain experiments, CT-26 cells were pre-incubated for 30 min with different concentrations (0.2–1 µM) of SB225002 and HT-29 cells were pre-incubated with different concentrations (0.1–1 µM) of Reparixin for 30 min. All migrated cells were counted microscopically in at least 5 different fields.

### Proliferation assay

Cell proliferation was evaluated in quadruplicates after staining CT-26 and HT-29 cells with calcein AM. Briefly, CT-26 and HT-29 cells were seeded in 96 wells culture plate at 5 × 10^3^ cells/well in media with or without CXCL2 (10–200 ng/ml) or CXCL8 (10–100 ng/ml) for 24 h (37 °C, 5% CO_2_), respectively. To assess proliferation, 2 µM of calcein AM was added per well and incubated for 1 h in dark at 37 °C, 5% CO_2_ and washed twice by ice cold PBS. Fluorescence was measured by use of a fluorometer (Tecan’s Infinite M200, Mannedorf, Switzerland) at excitation of 480 nm and emission of 530 nm and data expressed as a percentage of fluorescence intensity. In separate experiments, CT-26 and HT-29 cells were pre-incubated for 30 min with different concentrations of SB225002 (0.2–1 µM) and Reparixin (0.1–1 µM), respectively.

### Adhesion assay

Cancer cell adhesion to extracellular matrix proteins was evaluated using CytoSelect Cell Adhesion assay 48-well kit (Cell Biolabs, San Diego, CA) according to the manufacturer’s recommendations. In brief, CT-26 and HT-29 cancer cells were stimulated with CXCL2 and CXCL8, respectively, and allowed to adhere to different ECM proteins (vitronectin, fibronectin, collagen I, collagen IV, laminin I, fibrinogen and BSA) for 90 min. In separate experiments, CT-26 cells were pre-incubated with SB225002 1 µM or anti-mouse αV antibody 10 µg/ml and HT-29 cells were pre-incubated with Reparixin 100 nM for 30 min (37 °C, 5% CO_2_). Afterwards, CT-26 and HT-29 cells were stimulated with CXCL2 and CXCL8, respectively, and allowed to adhere to ECM proteins for 90 min. Cells were washed then lysed in lysis buffer and stained with the fluorescent CyQuant® GR Dye supplied with the kit. Cancer cell adhesion was measured by fluorometer (Tecan’s Infinite M200, Mannedorf, Switzerland) at 480 nm/520 nm and at least three times of experiments were performed.

### Experimental model of peritoneal cancer cell metastasis

All experimental procedures were performed in accordance to legislation on protection of animals and were approved by the Regional Ethical Committee for Animal Experimentation at Lund University, Sweden. Male Balb/c mice weighing 20 to 25 g were housed on an animal facility with 12–12 h light dark cycle at 22 °C and fed with a laboratory diet and water ad libitum. Mice were anesthetized with 7.5 mg ketamine hydrochloride (Hoffman-La Rhoche, Basel, Switzerland) and 2.5 mg of xylazine (Janssen Pharmaceutics, Beerse, Belgium) per 100 g body weight intraperitoneally. Mice were anesthetized, and a 1 cm long median incision to the abdominal cavity was performed. After laparotomy, 1 ml buprenorphine analgesia (0.05 mg/kg, Temgesic, Schering-Plough, NJ, USA) was administered subcutaneously to alleviate postoperative pain. Mice were then randomly assigned into five different groups; sham, vehicle + laparotomy, SB225002 + laparotomy, control IgG + laparotomy and anti-mouse αV antibody + laparotomy. CT-26 cells were detached from cell cultures using accutase and 98% viable cells were prepared for injection. 7 × 10^5^ viable CT-26 cells in 400 μl phosphate buffered saline (PBS) were mixed with vehicle, SB225002 (10 mg/kg), control IgG (1 mg/kg) and anti-mouse αV (1 mg/kg) antibody for 10–15 min prior to intraperitoneal administration. Animals received daily intraperitoneal injection of vehicle, SB225002 (10 mg/kg), control IgG (1 mg/kg) or anti-mouse αV (1 mg/kg) antibody for 10 days. Mice were examined daily for 10 days with no signs of distress, such as changes in appearance, respiration and physical activity. Upon experimental termination, mice were euthanized and tumor growth was evaluated by counting macroscopic tumor nodules in the peritoneal cavity.

### Quantitative real-time polymerase chain reaction (qRT-PCR)

Incisional wound tissues were collected in RNA later (Thermo Fisher Scientific, Rochester, NY, USA) and 50 mg tissue was homogenized in 1 ml Trizol (Sigma–Aldrich). Total RNA was isolated using Direct-zol RNA extraction kit (Zymo Research, Irvine, CA, USA) following the manufacturer’s instructions. The total RNA concentration and RNA purity of CT-26 cell lines were determined using Nano drop spectrophotometer at 260 nm absorbance. Reverse transcription was conducted with Revert Aid First Strand cDNA Synthesis Kit (Thermo Fisher Scientific) on 2.5 μg of total RNA in a final reaction volume of 20 μl according to the manufacturer’s instructions. Then, qRT-PCR was conducted in a final volume of 25 μl using SYBR Green dye (Clontech, Mountain View, CA, USA) for relative gene quantification. The RT-PCR program was as follows: initial denaturation (10 min at 95 °C) followed by 40 cycles of denaturation (30 s at 95 °C), annealing (1 min at 55 °C), and elongation (1 min at 72 °C). After the last cycle, a final extension of (1 min at 95 °C) was done. mRNA reference sequences were used to design primers using web-based primer design tools of the National Center of Biotechnology Information. The PCR primers used were as follows; CXCL2 sense 5′- AGAGGGTGAGTTGGGAACTA -3′, antisense; 5′- TACTCTCCTCGGTGCTTACA -3′, Beta actin sense; 5′-AGAGCCTCGCCTTTGCCGATCC-3′, antisense; 5′-CACATGCCGGAGCCGTTGTCG-3′. Expression of CXCL2 relative to beta actin was determined using 2^−ΔΔCT^ method.

### Statistical analysis

All statistical analyses were performed using GraphPad Prism® 8 software. For multiple comparisons Kruskal–Wallis One Way Analysis of variance on ranks followed by the Dunn’s post hoc test was used. P-value < 0.05 was considered significant. Mann Whitney test was used for comparison of two groups.

## Results

### Expression of chemokine receptors and their functional role in colon cancer cells

**Fig. 1:**
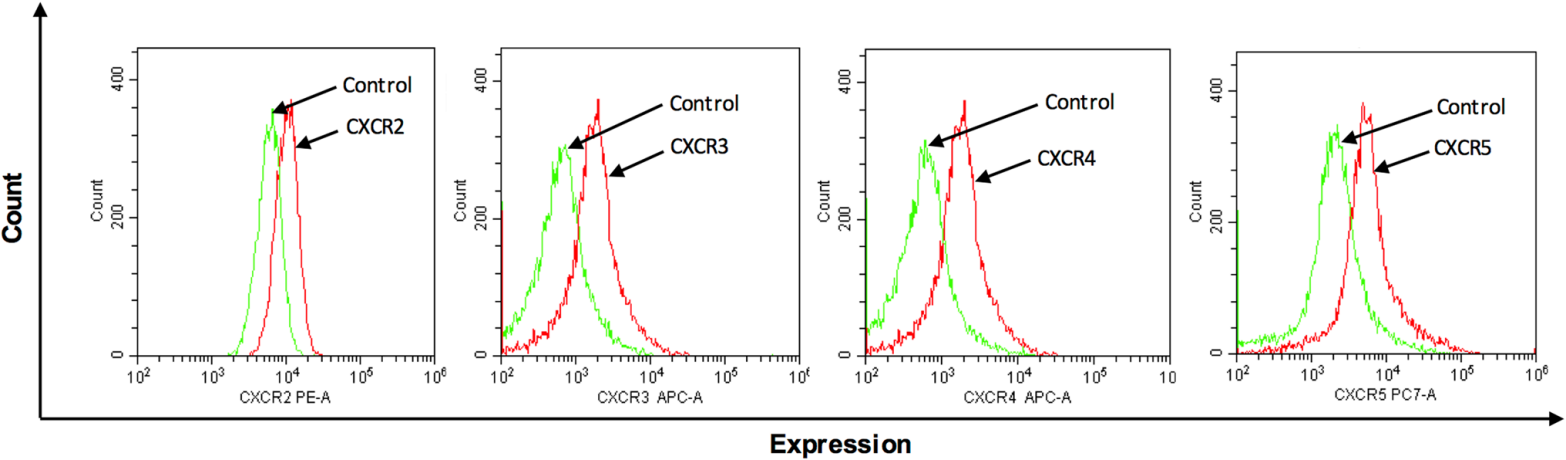
Chemokine receptor expression on colon cancer cells. Single-cell suspensions were prepared from confluent CT-26 cells and stained as outlined in Materials and Methods. Unstained cells were used as negative control and single tube staining used for each receptor. n = 4

**Fig. 2:**
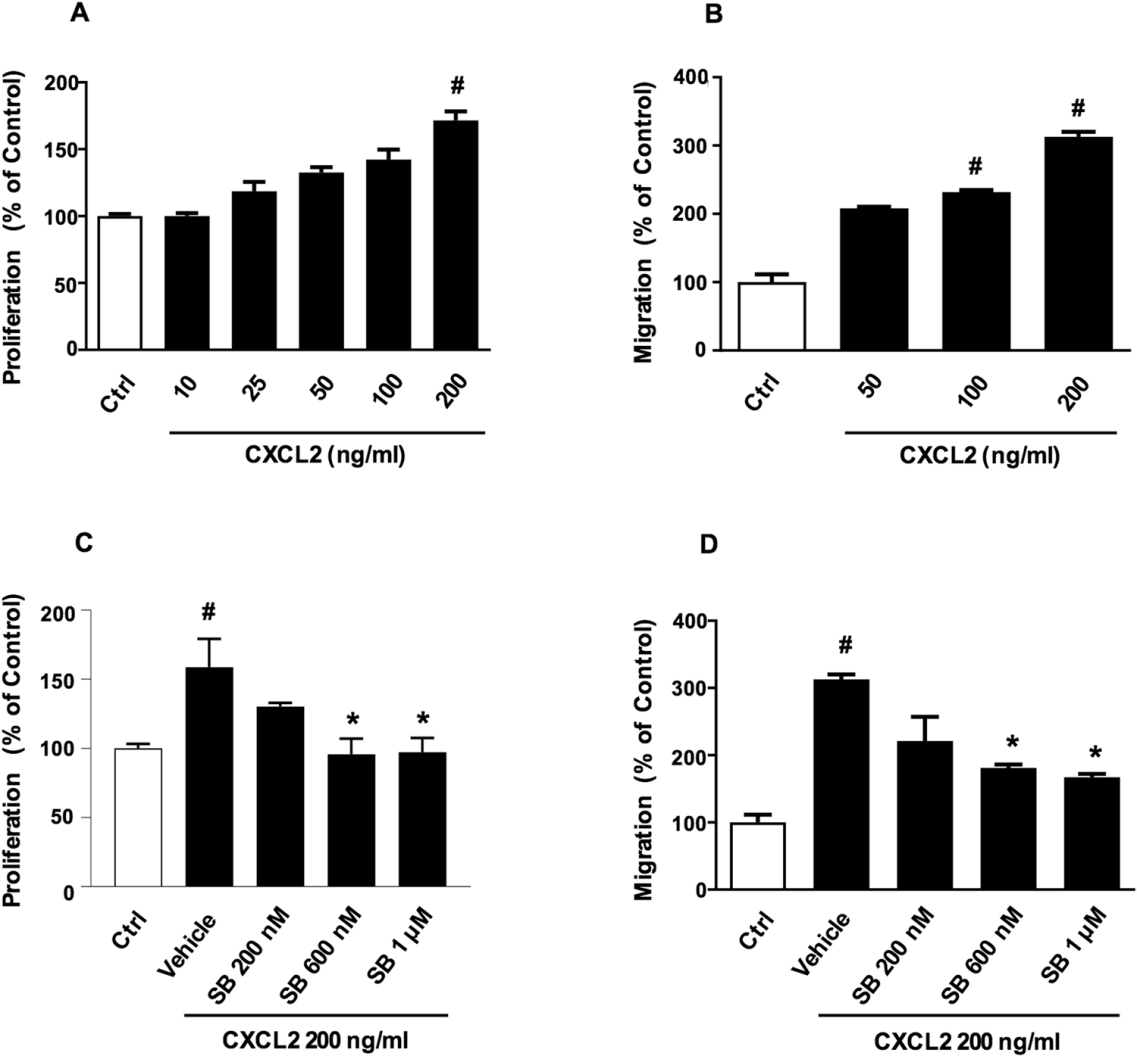
CXCL2 induces CT-26 colon cancer cell proliferation and migration. In vitro cell proliferation and migration in response to 24 h stimulation CXCL2 (10–200 ng/ml) without (**A**, **B**) or with (**C**, **D**) CXCR2 antagonist (SB225002) (0.2–1 μM). Proliferation was determined fluorometrically after adding calcein AM to cells as described in Materials and Methods. Migration was quantified by counting cells in High Power Fields in 5 different fields. Data represents mean ± SEM and n = 4. ^#^P < 0,05 vs Ctrl and *P < 0.05 vs Vehicle

Flow cytometric analysis of surface protein expression on CT-26 cells demonstrated clear-cut expression of CXCR2, CXCR3, CXCR4 and CXCR5 (Fig. 1), suggesting a potential role of CXC chemokines in colon cancer cell biology. Next, we examined the functional role of CXCR2 in CT-26 cells by studying the effect of the CXCR2 ligand CXCL2 on CT-26 colon cancer cell proliferation and migration. It was found that CXCL2 caused a dose-dependent increase in proliferation of CT-26 cells after 24 h of stimulation (Fig. 2A). For example, 200 ng/ml of CXCL2 increased CT-26 cell proliferation by 71% (Fig. 2A). Moreover, challenge with CXCL2 triggered significant colon cancer cell migration in vitro in a dose-dependent manner (Fig. 2B). Notably, CXCL2-evoked colon cancer cell proliferation and migration were dose-dependently attenuated by co-incubation with the CXCR2 antagonist SB225002 (Fig. 2C and D, respectively). For example, 1 µM of SB225002 reduced CXCL2-induced proliferation (Fig. 2C) and migration (Fig. 2D) by 40% and 46%, respectively.

### CXCL2-CXCR2 axis regulates peritoneal metastasis of colon cancer cells

**Fig. 3:**
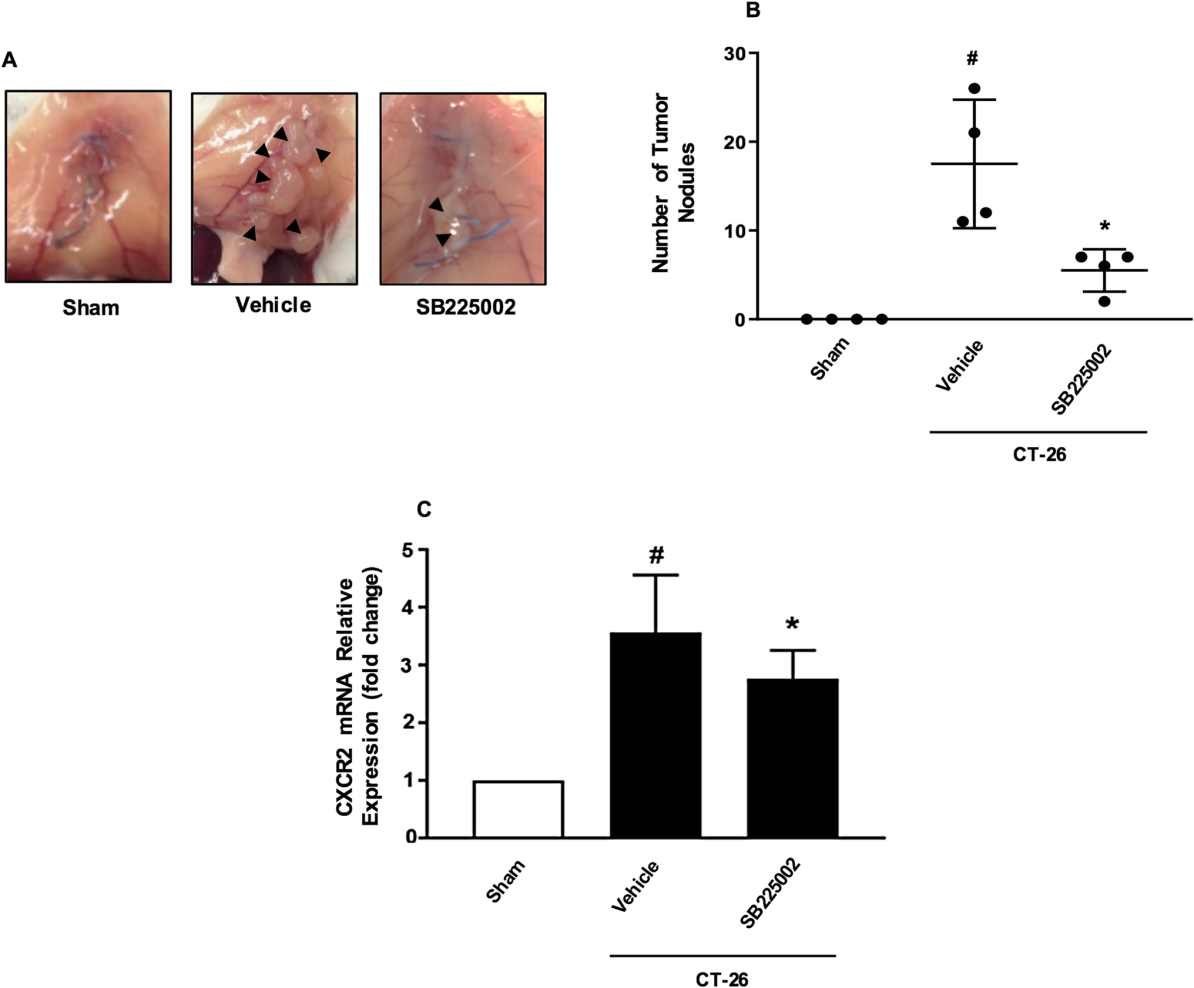
CXCR2 mediates peritoneal metastasis and binding to ECM proteins. CT-26 cells were injected intraperitoneally in laparotomized animals and mice received daily treatment with vehicle or the CXCR2 antagonist (10 mg/kg). After 10 days **A** the peritoneum was photographed and **B** the number of macroscopic tumor nodules was quantified in the peritoneum cavity. **C** Expression of CXCL2 mRNAs using qRT-PCR in tissue samples from the incisional line. Data represents mean ± SEM and n = 4. ^#^P < 0.05 vs Sham and *P < 0.05 vs Vehicle

In animals undergoing laparotomy, intraperitoneal administration of CT-26 cells caused multiple metastases along the midline incisional wound (Fig. 3A and 3B). In addition, laparotomy significantly increased mRNA expression of CXCL2 in the midline incision (Fig. 3C). Notably, pretreatment with the CXCR2 antagonist decreased the number of metastatic nodules by 70% in the peritoneal cavity in animals undergoing laparotomy (Fig. 3A and 3B).

### αVβ3 integrin and CXCR1 mediates colon cancer adhesion to ECM proteins

**Fig. 4:**
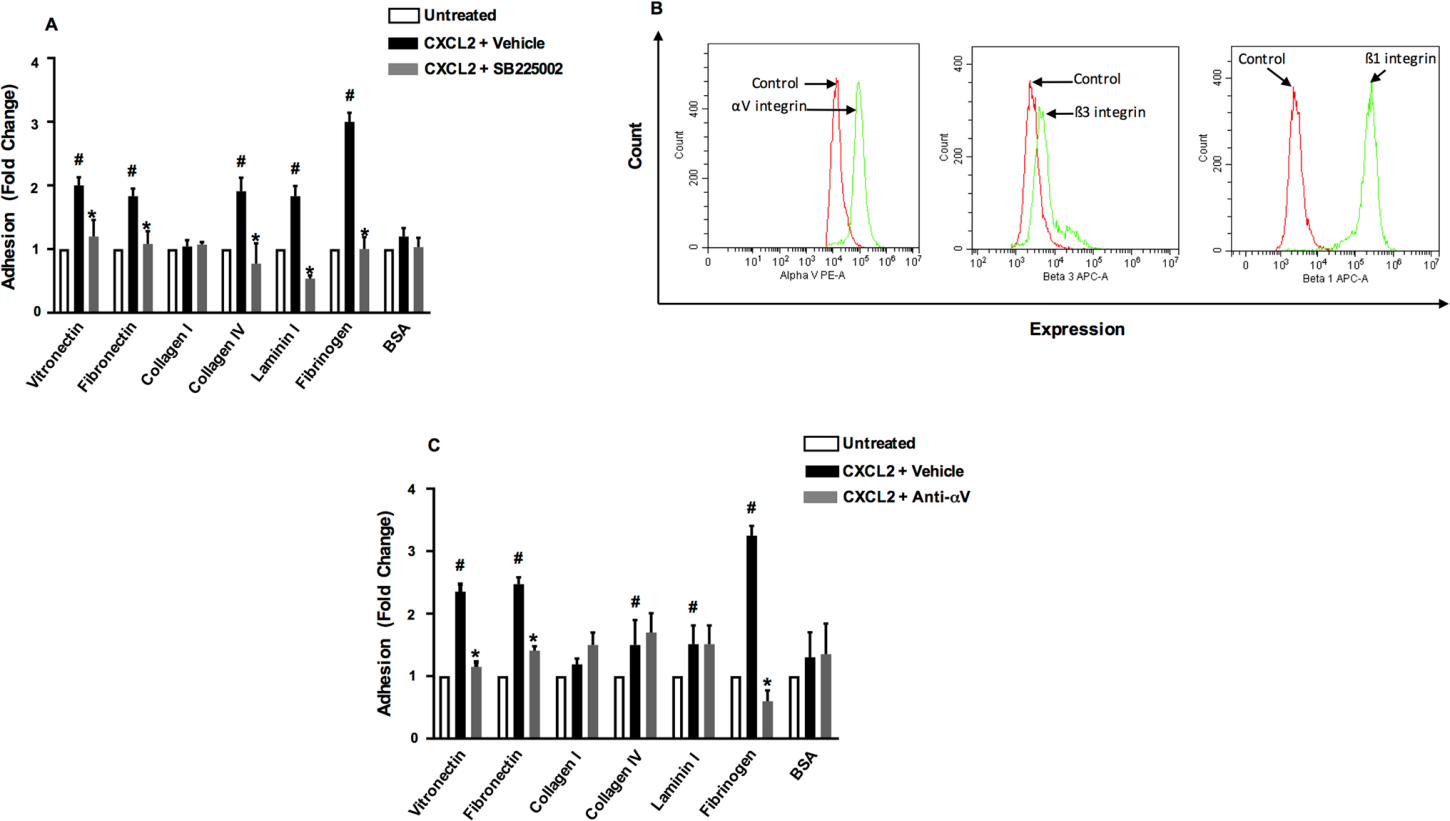
CT-26 cell adhesion and integrin expression. **A** CT-26 cells were stimulated with CXCL2 and allowed to adhere to wells coated with different ECM proteins as described in Materials and Methods. CT-26 cells were preincubated with vehicle or CXCL2 antagonist (1 μM). **B** Expression of αV, β1 and β3 integrin subunits on colon cancer cells. Single-cell suspensions were prepared from confluent CT-26 cells and stained as outlined in Materials and Methods. Unstained cells were used as negative control and single tube staining used for each receptor. **C** CT-26 cells were stimulated with CXCL2 and allowed to adhere to wells coated with different ECM proteins as described in Materials and Methods. CT-26 cells were preincubated with anti-CD51 (αV integrin) antibody 10 μg/ml. Data represents mean ± SEM and n = 4. **A** ^#^P < 0.05 vs untreated and *P < 0.05 vs CXCL2 + Vehicle. **B** ^#^P < 0.05 vs untreated and *P < 0.05 vs CXCL8 + Vehicle

We next asked whether stimulating CT-26 cells could increase colon cancer cell adhesion to ECM proteins. Indeed, it was found that co-incubation with CXCL2 enhanced CT-26 cell attachment to vitronectin, fibronectin, collagen IV, laminin I and fibrinogen (Fig. 4A). Interestingly, co-incubation of CT-26 cells with SB225002 abolished CXCL2-induced adhesion of colon cancer cells to vitronectin, fibronectin, collagen IV, laminin I and fibrinogen (Fig. 4A). Two of the most important receptors for several of these ECM proteins are the αVβ1 and αVβ3 integrins and we therefore examined whether CT-26 cells expressed these subunits. Indeed, it was found that CT-26 cells expressed the αV, β1 and β3 integrin subunits (Fig. 4B). To test the function of the αV integrin subunit, an antibody directed against αV integrin was co-incubated with the colon cancer cells. It was observed that immunoneutralization of αV abolished CXCL2-induced adhesion of CT-26 cells to vitronectin, fibronectin and fibrinogen but not to collagen IV and laminin I (Fig. 4C).

### αVβ3 integrin regulates peritoneal metastasis of colon cancer cells

**Fig. 5:**
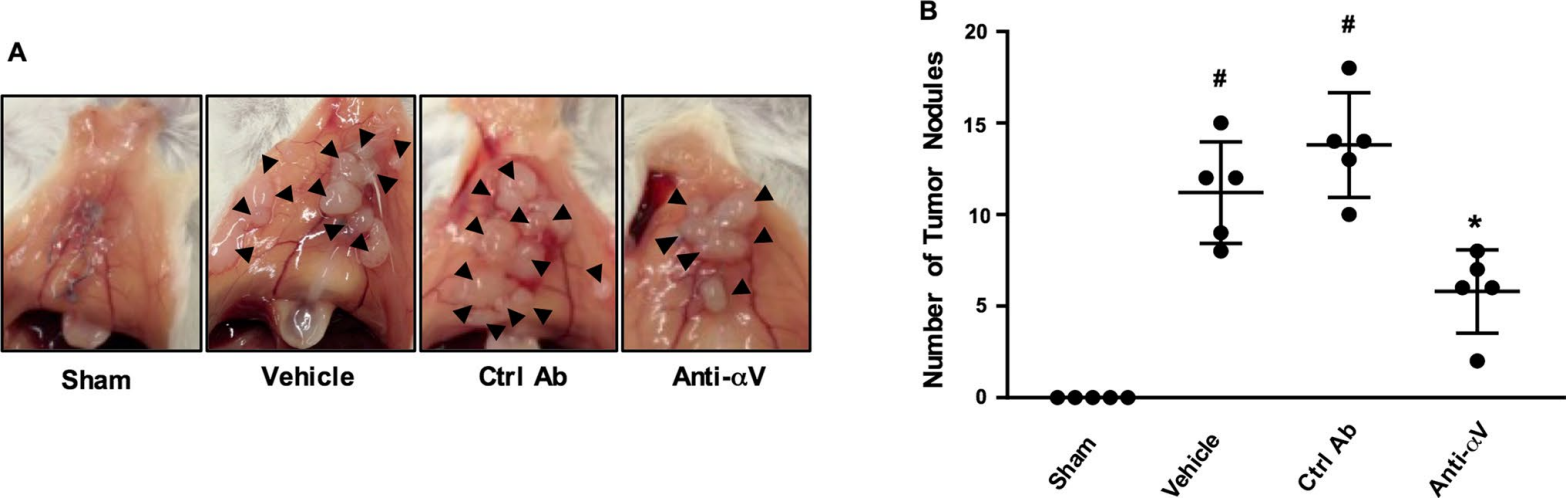
Role of αV integrins subunit in peritoneal metastasis of colon cancer cells. CT-26 cells were injected intraperitoneally in laparotomized animals and mice received daily treatment with a control antibody 1 mg/kg or an anti-CD51 (αV integrin) antibody 1 mg/Kg. **A** After 10 days the peritoneum was photographed and **B** the number of macroscopic tumor nodules were quantified in the peritoneum cavity. Data represents mean ± SEM and n = 5. ^#^P < 0.05 vs Sham and *P < 0.05 vs Ctrl Ab

We next asked whether the αV integrin subunit expressed on CT-26 cells might play a role in establishment of peritoneal metastases in vivo. It was found that administration of the antibody directed against αV integrin significantly decreased the number of metastatic nodules along the incisional line by 69% in laparotomized animals (Fig. 5A and B).

### Validation in human colon cancer cells

The murine experiments above were validated in a human colon cancer cell line HT-29 stimulated with CXCL8, which is the human homologue of the murine CXCR2 ligand [26]. In contrast to murine cells, human cells also express CXCR1 and CXCL8 is known to bind both CXCR1 and CXCR2 [27]. Flow cytometry revealed that HT-29 cells express CXCR1 but not CXCR2 or CXCR3 (Supplementary Fig. 1A). HT-29 cells also expressed αVβ3 (Supplementary Fig. 1A). Stimulation of HT-29 cells with CXCL8 dose-dependently increased proliferation (Supplementary Fig. 1B) and migration (Supplementary Fig. 1C). Moreover, co-incubation with the CXCR1/2 antagonist Reparixin significantly decreased CXCL8-induced HT-29 cell proliferation and migration (Supplementary Fig. 1D and 1E). In addition, CXCL8 stimulation increased HT-29 cell adhesion to fibronectin and laminin I, which was inhibited by co-incubation with Reparixin (Supplementary Fig. 2A). Moreover, co-incubation with an antibody against αVβ3 integrin decreased CXCL8-induced HT-29 cell adhesion to fibronectin and laminin I (Supplementary Fig. 2B).

## Discussion

Peritoneal carcinomatosis is an insidious aspect of colorectal cancer, especially as the growth and spread seem to be exaggerated by the trauma associated with surgical treatment. This study reveals basic mechanisms regulating migration, attachment and growth of colon cancer cells on the peritoneum in mice. Thus, our findings show that accumulation of colon cancer cells at peritoneal wounds is dependent on CXCR2 signaling. Moreover, these data suggest that colon cancer cell interactions with ECM proteins are dependent on αV integrins. Thus, this study defines several potential targets in reducing peritoneal dissemination of colon cancer cells.

Accumulating data suggest that chemokines play an important role in coordinating spread of malignant cells to the liver and lung [28]. Herein, it was found that the murine colon cancer cell line CT-26 expressed several of the pro-inflammatory chemokine receptors in the CXC chemokine family, including CXCR2, CXCR3 and CXCR4. The present study showed that CXCL2 induced robust proliferation and migration, underlining the important role of the CXC chemokines in colon cancer cell biology. Moreover, inhibition of CXCR2 dose-dependently decreased CXCL2-triggered migration and proliferation, suggesting that CXCL2 and CXCR2 interactions are operational in murine colon cancer cells. In this context, it is interesting to note that this CXCL2-CXCR2 axis has also been shown to be important in regulating colon cancer angiogenesis [18]. Thus, the CXCL2-CXCR2 axis might regulate several aspects in the establishment of peritoneal carcinomatosis in colon cancer cells. These findings were validated in a human colon cancer cell line (HT-29) showing that the human homologue of the murine CXCR2 ligand CXCL2 provoked proliferation and migration in a CXCR1/2-dependent manner in HT-29 cells, suggesting that our findings could be extrapolated to humans. This notion is also supported by a previous study reporting that damaged mesothelium can secrete CXCL8 [29].

In the present study, we observed that administration of the CXCR2 antagonist SB225002 reduced peritoneal metastasis along the incisional wound by 70%, indicating that CXCR2 expressed on colon cancer cells plays an important role in the development of peritoneal surface metastases. Notably, it was observed that CXCL2 was markedly upregulated along the incisional line in mice, which help explain the CXCR2-dependent accumulation of colon cancer cells at the peritoneal wounds. Moreover, CXCL2 is a powerful chemoattractant for neutrophils and a previous publication has shown that neutrophil extracellular traps can promote tumor cell metastasis to the liver [30]. In fact, a recent study suggested that neutrophil extracellular traps enhance peritoneal spread of gastric cancer cells [31]. Considered together, targeting the CXCL2-CXCR2 axis might inhibit colon cancer spread in the peritoneal cavity via several different mechanisms, i.e. on one hand decreasing cancer cell adhesion, migration and proliferation and on the other hand attenuating neutrophil recruitment and deposition of extracellular traps. This notion is also supported by a previous study showing that inhibition of CXCR2 by use of SB225002 also decreases peritoneal metastasis of ovarian cancer cells in a similar experimental model [32]. Nonetheless, our findings suggest that targeting chemokines is a new concept to counteract the establishment of peritoneal cancer growth. This concept is supported by a previous investigation showing that targeting CXCR4 reduces peritoneal metastases of ovarian carcinoma cells [33]. It should be noted that our findings show that CT-26 cells also express CXCR4, which could therefore be involved in the peritoneal dissemination of colon cancer cells and deserves to be examined in future studies. In this context, it should be mentioned that surgical trauma per se causes immune cell dysfunction which also contributes to increased spread of tumor cells in the peritoneal cavity [34].

It is generally held that cancer cells have a preferential ability to accumulate at surgical wound sites causing tumor recurrence [35–37]. One reason is that numerous growth factors and chemoattractants are formed in the wound that not only stimulate wound healing but also promote tumor cell adhesion and growth [9, 38]. Moreover, the ECM containing numerous different proteins with adhesive capacity is exposed to floating cancer cells at wound sites. Tumor cells that fail to adhere to ECM undergo anoikis [39] which makes adhesion a potentially effective target to inhibit peritoneal spread of colon cancer. Furthermore, most tumor cells circulating in the peritoneal cavity are rapidly removed by the immune system [40]. This concept is supported by the observation that the presence of free-floating cancer cells in the peritoneal cavity does not necessarily lead to peritoneal metastases [41, 42]. Herein, we observed that CXCL2 stimulation of CT-26 cells increased adhesion of colon cancer cells to vitronectin, fibronectin, collagen IV, laminin I and fibrinogen but not to collagen I. These findings were partially recapitulated in human cells showing that CXCL8 increased HT-29 cell adhesion to fibronectin and laminin I. Notably, flow cytometry revealed that both murine and human colon cancer cells express the αV and β3 integrin subunits. In addition, we observed that CT-26 also express the β1 subunit. This indicates that colon cancer cells can express both the αVβ1 and αVβ3 integrins, a notion that is in part supported by a previous study showing that human colon cancer cells express αVβ3 [43]. Interestingly, co-incubation of CT-26 cells with SB225002 abolished CXCL2-induced adhesion to these ECM proteins, suggesting that CXCL2-CXCR2 interactions activate αV integrins on colon cancer cells facilitating attachment to ECM proteins. In addition, we found that co-incubation with an antibody against integrin αV markedly reduced CXCL2-provoked adhesion of colon cancer cells to vitronectin, fibronectin and fibrinogen but not to collagen IV and laminin I, indicating that CXCL2-induced adhesion of colon cancer cells to certain ECM proteins is dependent on αV integrins. Similarly, we found that the CXCR1/2 antagonist decreased CXCL8-induced HT-29 cell adhesion to fibronectin and laminin I. Adhesive interactions between αV integrin subunit on one hand and several proteins in the extracellular matrix on the other hand could form a molecular basis for tumor cell adhesion to wound surfaces. Thus, we wanted to explore the role of the αV integrin subunit in colon cancer spread in the peritoneal cavity in laparotomized animals in vivo. It was found that immunoneutralization of integrin αV decreased the number of colon cancer nodules by 69%, suggesting that the αV integrin subunit constitute an important adhesion molecule for peritoneal metastasis of colon cancer cells. Considering that the laminin sequence YIGSR and the fibronectin amino acid sequence RGDS have been shown to attenuate peritoneal dissemination of gastric and ovarian cancer cells, respectively [44, 45], future studies should examine if ligands of the αV integrins could be useful to target colon cancer cell spread in the peritoneal cavity.

In conclusion, our results show that CXCR2 and αV integrins on colon cancer cells exert a key function in the formation of peritoneal metastases in vivo. Thus, blocking the CXCL2-CXCR2 axis or αV integrin adhesive interactions could be a useful strategy to reduce peritoneal recurrences after cytoreductive surgery of patients with peritoneal carcinomatosis.

### Supplementary Information

Below is the link to the electronic supplementary material.

Supplementary file1 (PPTX 166 kb)

## Data Availability

The raw data obtained and analyzed from this study are available from the corresponding author upon request.
