# Correction: CXCL2-CXCR2 axis mediates αV integrin-dependent peritoneal metastasis of colon cancer cells

**DOI:** 10.1007/s10585-024-10302-5

**Published:** 2024-07-27

**Authors:** Mattias Lepsenyi, Nader Algethami, Amr A. Al-Haidari, Anwar Algaber, Ingvar Syk, Milladur Rahman, Henrik Thorlacius

**Affiliations:** Section of Surgery, Department of Clinical Sciences, Malmö, Skåne University Hospital, Lund University, 20502 Malmö, Sweden

**Correction to: Clinical & Experimental Metastasis (2021) 38:401–410** 10.1007/s10585-021-10103-0

In Fig. 1 of this article. The CXCR3 representative image copied twice and mislabelled the x-axis and the correct Fig. 1 should have appeared as shown below.

**Fig. 1:**
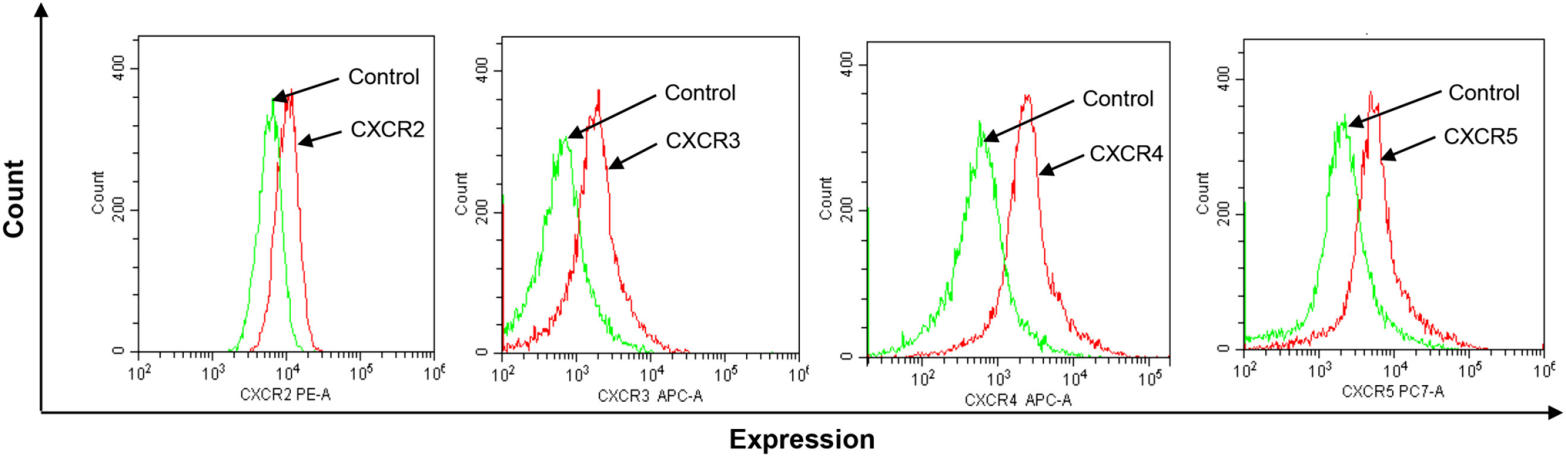
Chemokine receptor expression on colon cancer cells. Single-cell suspensions were prepared from confuent CT-26 cells and stained as outlined in Materials and Methods. Unstained cells were used as negative control and single tube staining used for each receptor. n = 4

