# Supplementary material for: CXCL2-CXCR2 axis mediates αV integrin-dependent peritoneal metastasis of colon cancer cells
Source: Clin Exp Metastasis. 2021 Jun 11;38(4):401–10. doi: 10.1007/s10585-021-10103-0 (PMC8318971; doi:10.1007/s10585-021-10103-0)
Supplement: Supplementary file 1 — Supplementary file1 (PPTX 166 kb) [file 10585_2021_10103_MOESM1_ESM.pptx]

## Slide 1
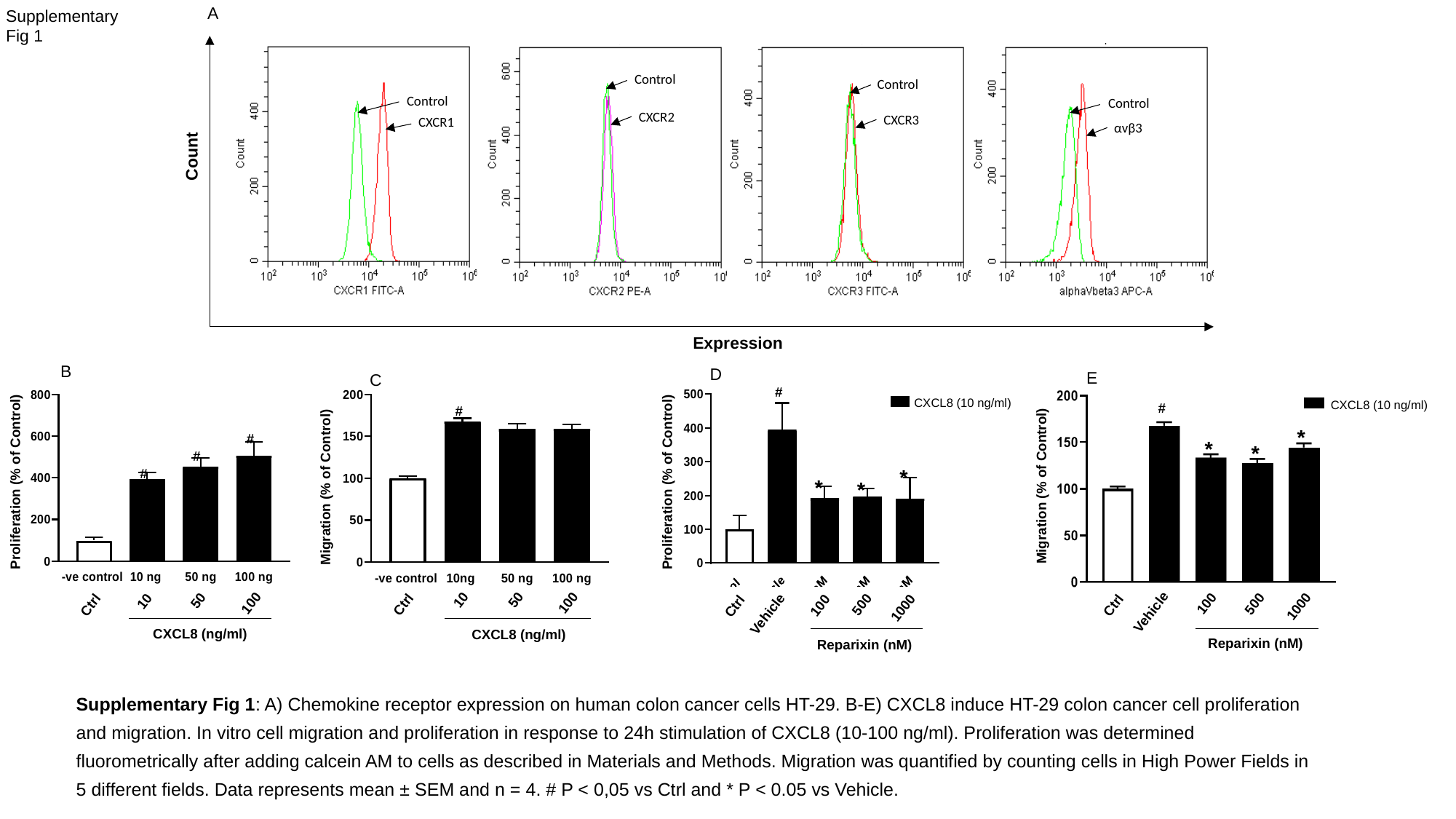

Supplementary Fig 1
A
Count
Expression
Control
Control
Control
Control
CXCR2
CXCR3
CXCR1
αvβ3
B
#
#
#
Proliferation (% of Control)
50
10
100
Ctrl
CXCL8 (ng/ml)
D
CXCL8 (10 ng/ml)
Proliferation (% of Control)
500
1000
100
Ctrl
Vehicle
Reparixin (nM)
#
*
*
*
E
CXCL8 (10 ng/ml)
Migration (% of Control)
1000
500
100
Ctrl
Vehicle
Reparixin (nM)
#
*
*
*
C
#
Migration (% of Control)
Ctrl
CXCL8 (ng/ml)
50
10
100
#
#
Supplementary Fig 1: A) Chemokine receptor expression on human colon cancer cells HT-29. B-E) CXCL8 induce HT-29 colon cancer cell proliferation and migration. In vitro cell migration and proliferation in response to 24h stimulation of CXCL8 (10-100 ng/ml). Proliferation was determined fluorometrically after adding calcein AM to cells as described in Materials and Methods. Migration was quantified by counting cells in High Power Fields in 5 different fields. Data represents mean ± SEM and n = 4. # P < 0,05 vs Ctrl and * P < 0.05 vs Vehicle.

## Slide 2
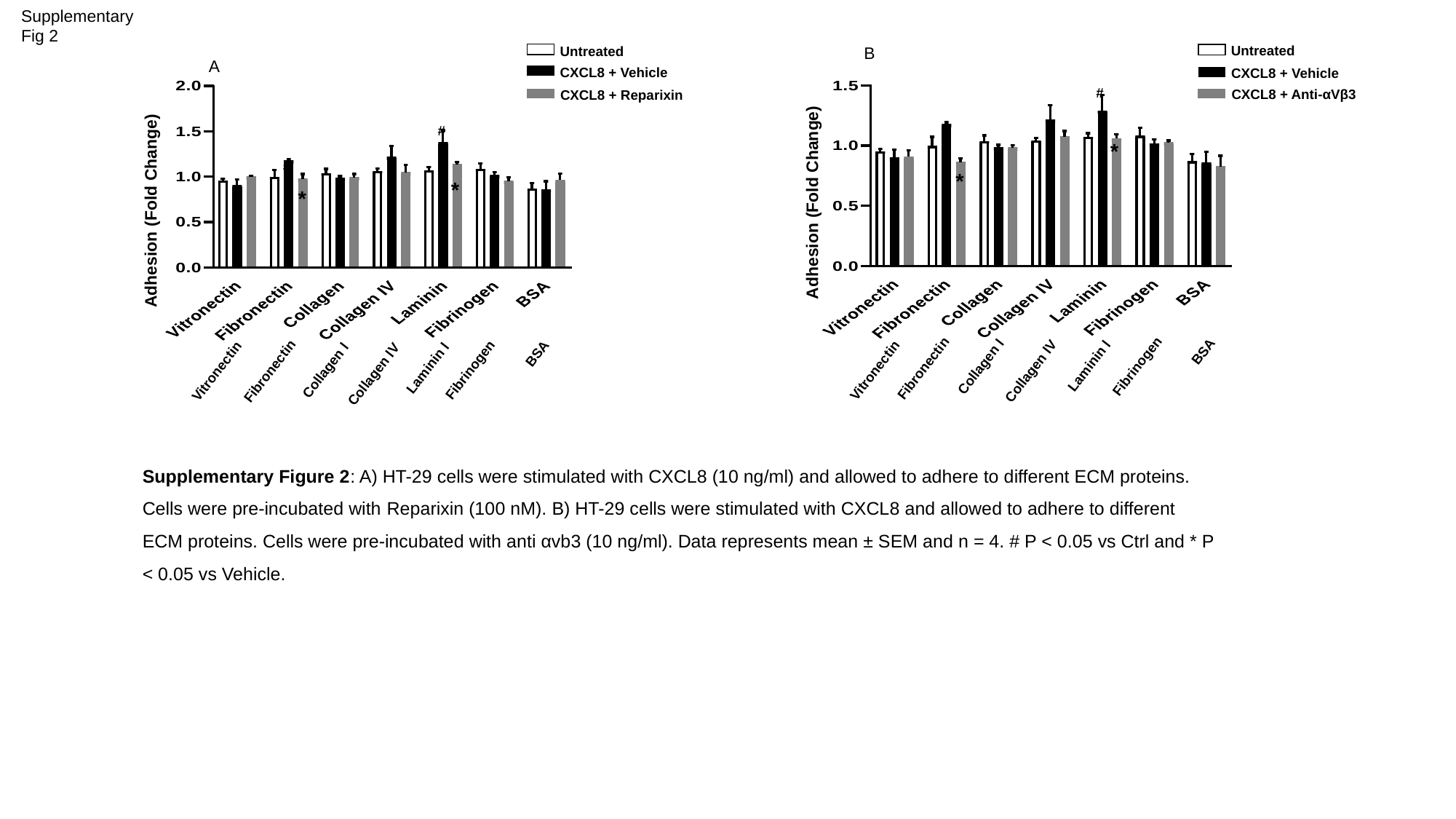

Supplementary Fig 2
Untreated
CXCL8 + Vehicle
CXCL8 + Anti-αVβ3
BSA
Fibrinogen
Collagen l
Laminin l
Vitronectin
Fibronectin
Collagen lV
#
#
*
*
Adhesion (Fold Change)
B
Untreated
CXCL8 + Vehicle
CXCL8 + Reparixin
Adhesion (Fold Change)
BSA
Laminin l
Vitronectin
Fibrinogen
Fibronectin
Collagen l
Collagen lV
#
#
*
*
A
Supplementary Figure 2: A) HT-29 cells were stimulated with CXCL8 (10 ng/ml) and allowed to adhere to different ECM proteins. Cells were pre-incubated with Reparixin (100 nM). B) HT-29 cells were stimulated with CXCL8 and allowed to adhere to different ECM proteins. Cells were pre-incubated with anti αvb3 (10 ng/ml). Data represents mean ± SEM and n = 4. # P < 0.05 vs Ctrl and * P < 0.05 vs Vehicle.
